# Acute Ischemic Stroke Treatment, Part 1: Patient Selection “The 50% Barrier and the Capillary Index Score”

**DOI:** 10.3389/fneur.2015.00083

**Published:** 2015-04-22

**Authors:** Firas Al-Ali, John J. Elias, Danielle E. Filipkowski, James E. Faber

**Affiliations:** ^1^Summit Neurovascular Specialists, Akron, OH, USA; ^2^Department of Research, Akron General Medical Center, Akron, OH, USA; ^3^Department of Cell Biology and Physiology, University of North Carolina School of Medicine, Chapel Hill, NC, USA

**Keywords:** acute ischemic stroke, patient selection, the 50% barrier, intra-arterial treatment, capillary index score, revascularization, stroke outcome

## Abstract

The current strategy for intra-arterial treatment (IAT) of acute ischemic stroke focuses on minimizing time from ictus to revascularization and maximizing revascularization. Employing this strategy has yet to lead to improved rates of successful outcomes. However, the collateral blood supply likely plays a significant role in maintaining viable brain tissue during ischemia. Based on our prior work, we believe that only approximately 50% of patients are genetically predisposed to have sufficient collaterals for a good outcome following treatment, a concept we call the 50% barrier. The Capillary Index Score (CIS) has been developed as a tool to identify patients with a sufficient collateral blood supply to maintain tissue viability prior to treatment. Patients with a favorable CIS (*f* CIS) may be able to achieve a good outcome with IAT beyond an arbitrary time window. The CIS is incorporated into a proposed patient treatment algorithm. For patients suffering from a large stroke without aphasia, a non-enhanced head CT should be followed by CT angiography (CTA). For patients without signs of stroke mimics or visible signs of structural changes due to large irreversible ischemia, CTA can help confirm the vascular occlusion and location. The CIS can be obtained from a diagnostic cerebral angiogram, with IAT offered to patients categorized as *f* CIS.

## Introduction

The current strategy for acute ischemic stroke (AIS) treatment is based on two pillars: time from ictus to revascularization (TIR) and revascularization success as measured by the modified thrombolysis in cerebral ischemia scale (mTICI). The assumption is that clinical outcome following AIS is dependent on the interaction of these two factors. The shorter the TIR and the higher the mTICI, the better the outcome. It follows that the strategy behind current intra-arterial treatment for acute ischemic stroke (IAT-AIS) is the faster and more complete the revascularization, the better the clinical outcome. However, despite the recent impressive improvement in revascularization rates and decrease in time to revascularization, until recently the clinical improvement rate remained unchanged at approximately 40–45% (Table 1) with a ratio of good clinical outcome (GCO) in treatment vs. control arms of approximately 1.7 (1–11). Recent trials have published GCOs above 50% in the treatment arm, but with the same ratio of GCOs between the treated and untreated arms around 1.7 (12, 13). How we can explain this consistency? A fresh look at our strategy and selection criteria is obviously warranted.

**Table 1:** **Clinical outcomes across IAT-AIS trials**.

Trial	% mRS 0–2 (3 months)	Time to IAT (h)	% TIMI 2, 3
PROACT II	42.3^a^	4.5^b^	58
IMS I	43	3.05 ± 0.8^b^	56
IMS II	46	n/a	64
IMS III	40.8	3.5^b^	81^d^
SYNTHESIS	41.9	3.45^c^	n/a
SWIFT	37	4.9^b^	83
TREVO 2	39.9	4.7^c^	90
MR CLEAN	32.6	4.3^c^	58.7^d^
EXTEND-IA	71	3.5^c^	86^d^
ESCAPE	53	3.1^c^	72.4^d^

*^a^Barthel Index 9 and 10*.

*^b^Mean*.

*^c^Median*.

*^d^TICI 2, 3 for M1 occlusion*.

## Physiological Background and the 50% Barrier

Normal cerebral blood flow (CBF) is 50–55 ml/100 g/min (14, 15). AIS induces a rapid and sustained reduction in CBF. Clinical signs of ischemia generally become apparent when CBF drops below 23 ml/100 g/min (16). If residual CBF (rCBF) further decreases to 15–16 ml/100 g/min, the cortical-evoked potential ceases within seconds (16). The rate of depression of the evoked potential (EP) amplitude (expressed in units of percent of control/min) is highly correlated with the residual flow, following a linear relationship with the regression line intercepting the flow axis at 15.2 ml/100 g/min (17). The data strongly suggest a threshold-like relationship also exists between the amplitude of the EP and local blood flow. If flow is greater than approximately 16 ml/100 g/min the EP is not affected, but at flows less than approximately 12 ml/100 g/min the EP is abolished (17). Neither the clinical signs of ischemia nor cessation of the EP is synonymous with cell death, but cessation of the EP is one of the final stages before irreversible injury (infarction). Its physiological purpose is to conserve energy by decreasing cell metabolism to the minimal level possible; however, cell death ensues thereafter.

**Figure 1:**
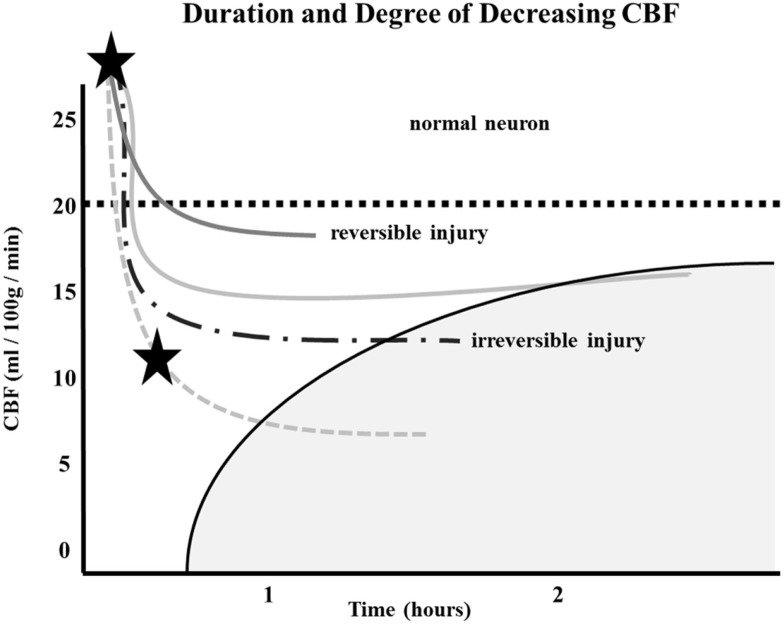
**Depth of ischemia and time to irreversible cerebral damage: time to irreversible cerebral damage depends on the depth of ischemia, which depends on the pial collateral supply to the ischemic territory**. Since different patients have different collaterals, the depth of ischemia will vary among patients, as will the time available for therapy to salvage the tissue (16).

Similarly, the relationship between time to irreversible damage and rCBF is well-documented (18). In one study, rCBF in monkeys was measured in the ischemic area with time after occlusion until irreversible tissue damage occurred (16). An infarction threshold was observed relating the rCBF to time between the initial drop in CBF to irreversible ischemia (Figure 1). This work confirmed prior studies using the neuronal EP and showed that when rCBF reached a low level of around 10 ml/100 g/min, the available time to salvage the brain tissue was extremely short (<1 h) (16).

The depth of ischemia, i.e., the level of rCBF, will vary from patient to patient depending on the available retrograde pial collaterals to the ischemic area. The major determinants of the amount of collateral perfusion are the number and diameter of these pial collaterals, plus perfusion pressure and resistance above and below the collateral network. Greater collateral numbers and diameters sustain a higher rCBF, thus more salvageable brain and a smaller final infarct volume.

Following AIS, rCBF stays virtually unchanged if spontaneous recanalization of the occluded blood vessels does not occur (16, 18, 19). While the clinical symptoms of ischemia will often resolve if CBF is restored promptly, prolonged low levels of rCBF leads to irreversible brain tissue damage. Since the time of ischemia that the brain tissue can tolerate before irreversible damage ensues depends on the rCBF value, which is patient-specific and highly dependent on the collaterals, it follows that *every patient has his or her own time* (Figure 1) (16, 18, 19). Hence, if we correctly select patients that are optimal candidates (patients with ischemic but viable tissue) and are able to achieve safe, full, and timely revascularization (prior to irreversible ischemic damage occurring), the clinical symptoms of a stroke should improve significantly and rather quickly.

**Figure 2:**
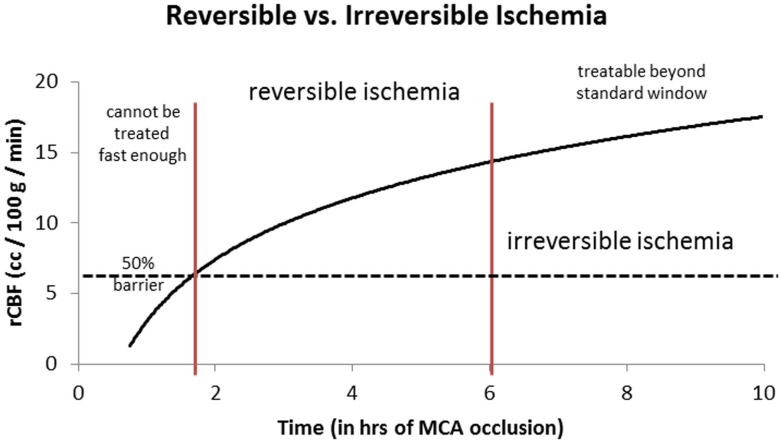
**Logarithmic time curve: the infarction threshold distinguishing between reversible and irreversible ischemia as a function of rCBF and time from ictus**. Time window here is an approximation. The vertical lines are an approximation and have not yet been validated (20).

Given this information, the most logical explanation for the remarkably consistent results of the different IAT-AIS trials, with <50% GCOs (modified Rankin Score, or mRS, ≤2), is that around half of treated patients have poor pial collaterals, thus causing them to have a relatively low rCBF such that they enter into irreversible ischemia *before* therapy can be administered, even when *timely* (within 6 h) revascularization is achieved. This observation implies a potential ceiling effect for IAT-AIS; we call this phenomenon *the 50% barrier* (Figure 2).

## The Genetic Factor?

Why is there such variability in collateral-dependent flow in patients with AIS, as exemplified by the above and many other studies? (21) Could the number and diameter (i.e., extent) of cerebral collaterals vary among individuals? While we do not have answers for humans yet, recent studies in mice suggest the answer may be yes and that genetic background may be important. In mice, pial collaterals form late in gestation, after the cerebral artery trees are well-established (22, 23). Likewise in humans, the middle cerebral artery (MCA) tree is already well-established by 9 weeks gestation, with pial collaterals beginning to appear by 14 weeks (24, 25). Collateral formation occurs by a unique process, termed collaterogenesis, that differs significantly from development of the general arterial–venous circulation; moreover, this process determines the collateral extent present in the adult (22, 23). Interestingly, naturally occurring differences in genetic background, which have no discernible effect on formation of the general circulation or its extent and function in the adult, have profound effects on collaterogenesis (23, 26). Thus, the extent of the pial collaterals in the neocortex varies by 56-fold among 21 mouse strains with different genetic backgrounds, resulting in a 30-fold variation in infarct volume after MCA occlusion (27–29). A single polymorphic locus on chromosome 7, denoted, “Determinant of collateral extent-1 (*Dce1*),” has been identified as *causal* for more than 80% of this variation, as exemplified in the two index strains (30). In that study, congenic methods were used to replace the at-risk allele of *Dce1* in the strain with poor collaterals with the allele from the strain with abundant collaterals. This restored the poor collateral phenotype to nearly that in the good strain, i.e., 83% correction of low collateral extent, and – after MCA occlusion – a 4.5-fold increase in blood flow in the territory at risk and 85% reduction of final infarct volume. Thus, ischemia and infarct volume were strongly reduced by exchanging a single genetic locus (30). These findings demonstrate that the *Dce1* locus harbors a critical link in the pathway that controls collaterogenesis. Although the causative genetic element(s) at *Dce1* is not yet known, several candidate genes have been identified (30). Since the pathways that control vascular development in the embryo are highly conserved among vertebrates, the same or a closely related gene(s) is likely to contribute to the wide variation in collateral status in humans. A prospective multi-center study, “Genetic Determinants of Collateral Status in Stroke (GENEDCSS) has been initiated to test this hypothesis (31). This study will determine if variation in collateral score, stroke severity, functional recovery, and other outcomes are linked to a polymorphism(s) at human *Dce1* and/or at several related candidate genes in patients with acute MCA stroke.

One’s genetic background may not be the only factor that causes variation in collateral extent. Environmental factors also cause collateral insufficiency, at least in mice, although the magnitude of their impact has thus far not approached that of genetic background. Thus, aging (32), other cardiovascular risk factors such as hypertension, metabolic syndrome, and diabetes (33), as well as endothelial dysfunction *per se* (34), cause loss of pial collaterals and reduced diameter of those that are still present (collateral rarefaction). This rarefaction is accompanied by substantial increases in infarct volume after MCA occlusion. These findings have recently found support in patients with AIS(21).

It is also important to note that variation in the extent of the anterior communicating artery (ACom) and posterior communicating artery (PCom) collaterals is well-known to exist in humans, including those with acute stroke. The contributions of genetic, environmental, and stochastic factors to this variation are unknown, although they are currently under investigation in mice (JE Faber, personal communication). Moreover, the extent to which such variation combines with variation in pial collaterals to impact rCBF remains to be determined.

How might identification of a “collateral gene” like *Dce1* in mice benefit patients with acute stroke? A biomarker for collateral extent would provide a rapid point-of-care test to aid imaging methods, used during stroke triage to measure collateral status (e.g., conventional angiography and CT/MR perfusion), to help tailor the time-window for treatment with intravenous and/or endovascular recanalization therapies. A genetic marker of collateral abundance would also help stratify patients to reduce the presumed large contribution of collateral differences to the variability seen in past trials, and help more accurately assess the merit of the treatment used (e.g., intra-venous vs. intra-arterial vs. embolectomy). Identifying a risk allele for collateral insufficiency in humans would also be prognostic, adding to our understanding of why some patients do worse than others. Eventual identification of the causal gene(s) at *Dce1* may also provide therapeutic targets aimed at the collateral circulation for future development. Healthy individuals carrying the risk polymorphism could be encouraged to adopt lifestyles and treatments to avoid acquiring risk factors for cardiovascular disease and stroke that have been found in animal studies (22, 30, 31) [with support coming in human studies (4)] to cause progressive loss of collaterals and increased severity of stroke.

## Patient Selection

### Current imaging selection tests: Non-invasive neuroimaging

#### Diffusion MRI

Diffusion MRI is the best available method for the early detection of infarct core (35–38). Acute infarction produces a high contrast abnormality on diffusion-weighted images (DWI), the volume of which is relatively simple to quantify (39). The high contrast-to-noise (CNR) ratio of DWI makes it accurate. DWI abnormalities sometimes reverse (40), but this is rare (41) and when it occurs it usually involves only a small part of the lesion (42). Additionally, a DWI reversal is often a pseudo-reversal in that such tissue proceeds to infarction despite apparent temporary normalization of the DWI signal abnormality (42).

Studies have shown that a DWI abnormality volume of >70 ml is highly specific for a poor outcome (43, 44), and that this threshold volume is useful in selecting patients for endovascular intervention (45, 46). This threshold was successfully employed in the Diffusion and Perfusion Imaging Evaluation for Understanding Stroke Evolution Study II (DEFUSE II) trial (42). The use of early infarct “core” identification for triage decisions is supported by the observations that the final infarct volume is the single best predictor of good outcome at 90 days (39, 40). As has been shown (47), good outcomes are observed in nearly half such patients when the final infarct volume is 60 ml or less. The rate of good outcomes rapidly declines with infarcts that are larger. The use of a 70 ml DWI volume threshold (48) to successfully guide endovascular treatment was recently independently verified in a study at the Cleveland Clinic (49).

#### CT

CT is a front-line imaging modality for acute stroke because it is reliable for detecting hemorrhage. Moreover, CT angiography (CTA) may be subsequently acquired. However, non-contrast CT is unreliable for detecting the early infarct core (50, 51). It is highly specific for infarction when a hypodensity is clearly visible, but such changes typically occur late.

#### CT Perfusion

Much research has been devoted to developing CTP techniques for identification and quantification of the early infarct core. However, it is not sufficiently reliable for this purpose. This is because it is a method that has inherently low signal-to-noise ratio (SNR) and CNR, producing “noisy” images with high measurement error (46, 52). Proponents of CTP may have been misled by correlation and regression studies of CTP-derived parameters in comparison to DWI or another gold standard. These studies typically show statistically significant correlations. Some investigators extrapolate a high correlation in a population of measurements to high accuracy of the measurement in an individual. This is not valid (53). A recent evidence-based analysis of diffusion and perfusion imaging in stroke by the Therapeutics and Technology Assessment Subcommittee of the American Academy of Neurology found that diffusion MR was a Level A/Class I method, but found insufficient evidence to even classify perfusion imaging (37). Furthermore, CTP typically only encompasses a limited number of slices and often fails to capture even half the tissue volume at risk for infarction that one is interested in determining infarction volume in.

There is no consensus on how to best apply CTP. A variety of acquisition parameters have been used, as well as many different data processing methods. Additionally, different parameters (e.g., cerebral blood volume, CBV, or CBF thresholds) have been proposed for defining infarcted tissue (54). It is thought that standardization and validation will make CTP viable (53). However, CTP is unlikely to become a reliable method (46, 52). Theory informs us that CBV may be elevated or depressed in core tissue and thus it is not useful. This has been empirically confirmed (55). CBF is more capable of estimating the infarct core. The reasoning is that below a certain CBF threshold, brain tissue is very likely to be viable only for a short period of time. However, there are major problems that are related to the underlying imaging physics: the CNR of infarct cores on CTP-derived CBF images are very low (52). At its current state, the errors in CTP-derived estimates of CBF are too high to be used to reliably guide treatment in an individual patient with a severe anterior circulation stroke.

### The limitations of non-invasive testing

Despite its promise, the merit of any non-invasive imaging test in patient selection for AIS treatment has yet to be proven in a multicenter, prospective, randomized clinical trial. Furthermore, the issue of what is considered acceptable sensitivity, specificity, and positive predictive value of these screening tests has not yet been addressed.

In a recent paper, Alberta Stroke Program Early CT (ASPECT) score was not found to help in patient selection in AIS to predict outcome (56). Even MRI diffusion and perfusion imaging did not demonstrate a strong enough positive predictive value where only approximately half of patients with AIS, who were selected for endovascular treatment, achieved GCO following successful revascularization (42, 49). We believe that the low positive predictive value (50%) of these tests is due to the inability of different non-invasive tests to distinguish between normal and ischemic (but viable) cerebral tissue on one hand, and its inability to distinguish between ischemic tissue and irreversible ischemia early on, on the other hand, due to the time delay needed for the structural changes of cerebral infarction to become readily apparent (42, 49, 56, 57).

For a screening test to be a useful patient selection tool, it must be highly correlated to the functional clinical outcome. In our opinion, existing non-invasive tests do not meet this requirement. This relatively low positive predictive value of the different imaging techniques used has multiple implications. First, it hinders our ability to develop an accurate prognosis for the patient and his or her family. Second, we may proceed with a costly treatment without benefit (futile recanalization). In some cases, the patient may experience worsening clinical symptoms due to increasing the cerebral injury by reperfusion-mediated vasogenic edema, or perhaps even hemorrhagic transformation by forcing blood into the infarcted area (harmful revascularization). Finally, and more importantly, we may deny the treatment to patients based on an artificial time window, for whom IAT may still be beneficial. Increasing the accuracy of patient selection is clearly needed.

## The Capillary Index Score

The role of collaterals in improving clinical outcome in patients with AIS is now widely accepted (58–60). Recently, using the American Society of Interventional and Therapeutic Neuroradiology/Society of Interventional Radiology (ASITN/SIR) collateral score (58), the Interventional Management of Stroke (IMS) III investigators were able to confirm the previous reports on the positive effect of better collaterals on revascularization and clinical outcome (59).

**Figure 3:**
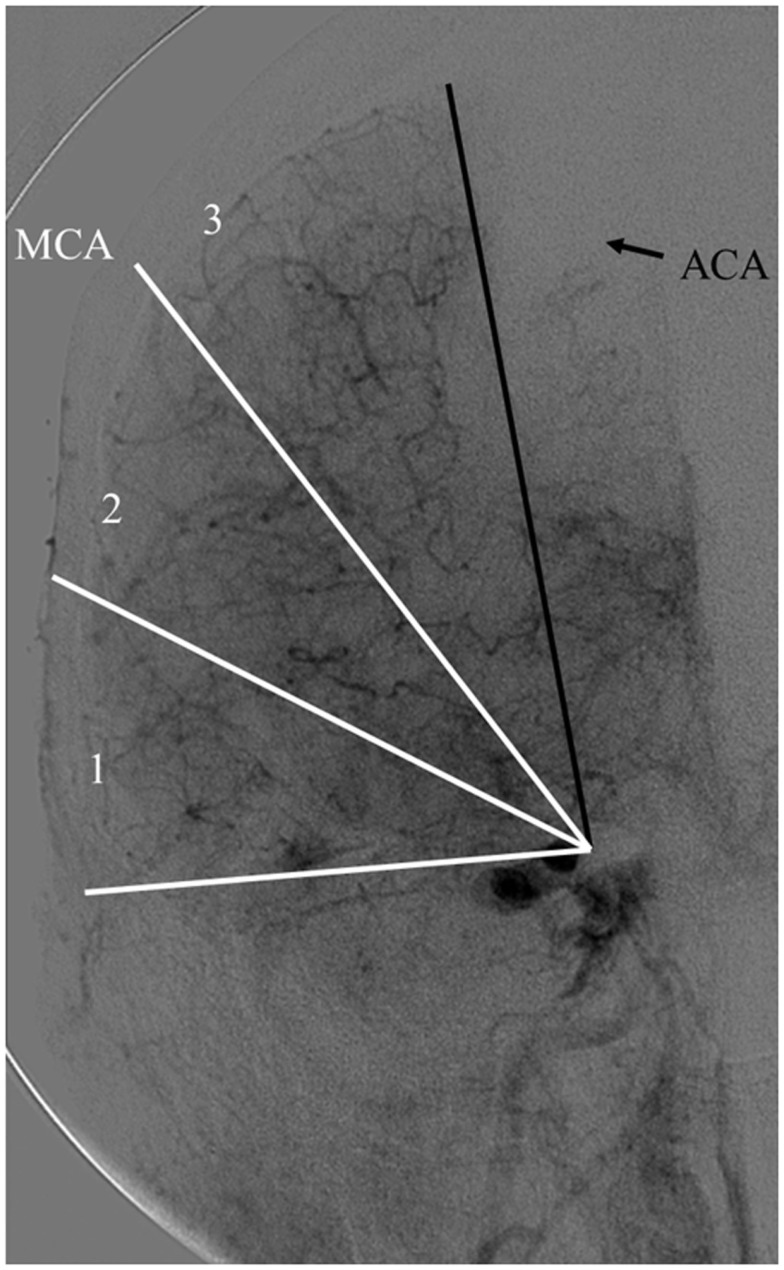
**Calculating the capillary index score (CIS)**. A frontal view of normal diagnostic cerebral angiogram. The territory of the middle cerebral artery (MCA) is being used as an example of an ischemic territory. The ischemic territory is divided into three equal sections; each section is given a 1 if it exhibits capillary blush, or a 0 if no capillary blush is present. The CIS is the sum of these three numbers. CIS can range from a score of 0 to 3 (20, 61).

**Figure 4:**
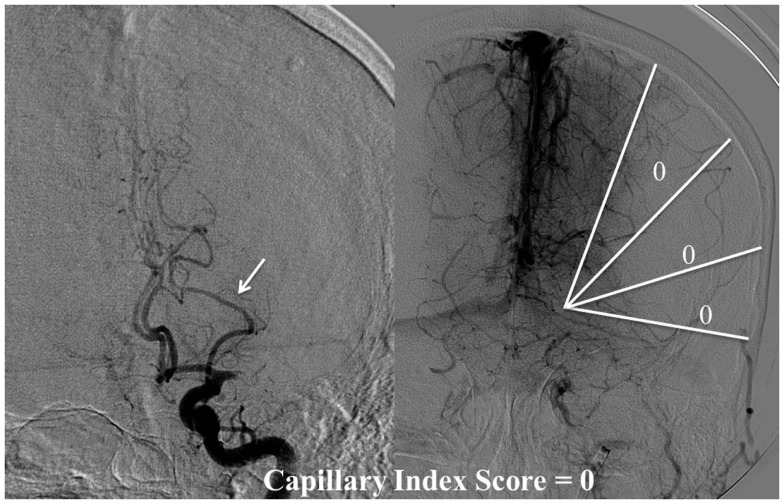
**CIS = 0**. In this patient with proximal left middle cerebral artery occlusion, we can calculate the CIS from this injection only since the only other potential collateral to the MCA territory is from the left posterior cerebral artery (PCA), which is filled in the injection through the posterior communicating artery (Pcom, arrow). If we divide the ischemic territory (Lt MCA territory) into three sections, none of these sections exhibit a capillary blush, late in the venous phase; therefore, the CIS = 0.

**Figure 5:**
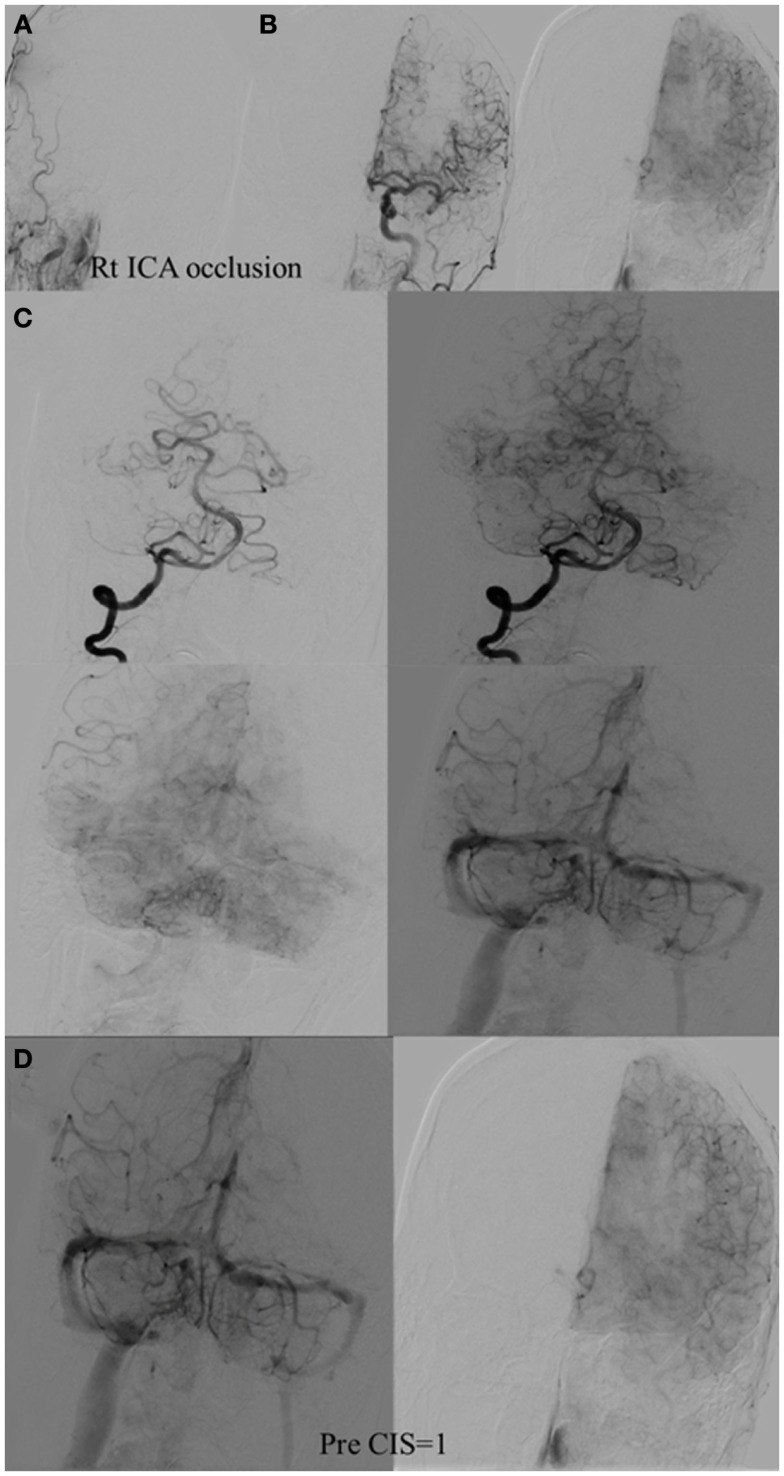
**CIS = 1**. **(A)** Occlusion of intracranial right ICA. **(B)** Injection of the left ICA demonstrates absence of the Acom; hence, no cross filling to the right hemisphere from this injection (ischemic territory = right middle and right anterior carotid arteries). **(C)** Injection of the right vertebral artery demonstrates partial opacification of the temporal and parietal lobes through the right PCA via pial collaterals. **(D)** Delayed combined venous phase of the left internal carotid and right vertebral showing only one-third of the ischemic territory (right middle cerebral and interior cerebral arteries) territory demonstrates capillary blush. CIS = 1.

**Figure 6:**
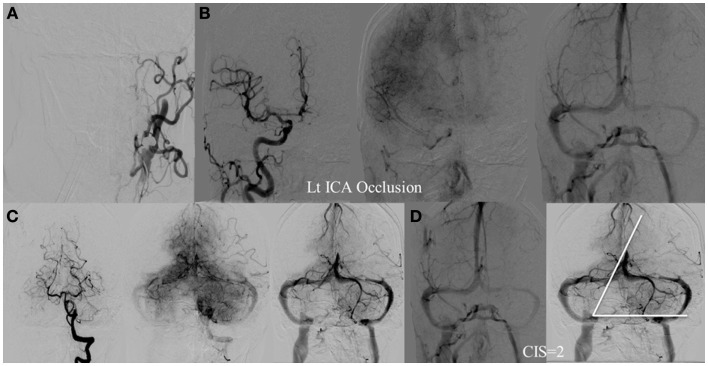
**CIS = 2**. **(A)** Occlusion of the left ICA. **(B)** Injection of the right ICA demonstrates filling of the left ACA territory through the Acom with partial opacification of the fronto–parietal lobes via pial collaterals. **(C)** Injection of the left vertebral artery demonstrates partial opacification of the left temporal lobes via pial collaterals. **(D)** Delayed venous phase of the right ICA and left vertebral showing approximately two-third of the ischemic territory (left middle MCA) to demonstrate capillary blush.

**Figure 7:**
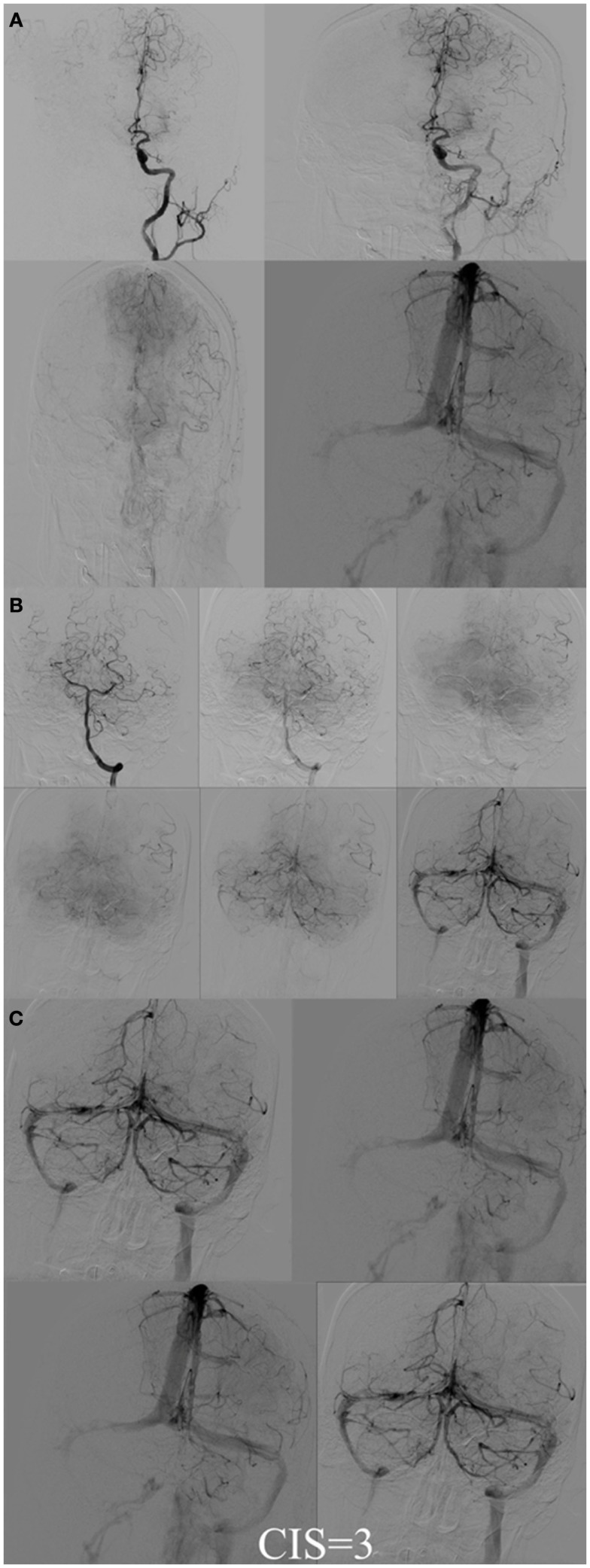
**CIS = 3**. **(A)** Injection of the left ICA demonstrates occlusion of proximal left MCA (ischemic territory = left MCA) with partial opacification of the left fronto-parietal lobes via pial collaterals of the left ACA. **(B)** Injection of the left vertebral artery demonstrates partial opacification of the left temporal lobes via pial collaterals. **(C)** Delayed venous phase of the left ICA and the left vertebral artery. All the ischemic territory (left MCA) demonstrates capillary blush. CIS = 3.

The Capillary Index Score (CIS) was first introduced from the Borgess Medical Center-Acute Ischemic Stroke Registry (BMC-AIS Registry) data (61) with the aim to improve the criteria for patient selection in AIS. The presence of capillary blush was proposed to be a *marker of residual viable tissue*, with its absence implying irreversible ischemia. The CIS is a simple 4-point scale ranging from 0 to 3. The ischemic territory on the frontal view of a diagnostic cerebral angiogram (DCA) is divided in three equal segments (Figure 3). If a segment does not demonstrate a capillary blush it is assigned 0 points, whereas it is assigned 1 point if it exhibits capillary blush. The final CIS is the sum of these three segmental scores (Figures 4–7). Therefore, CIS of 0 means no angiographic capillary blush was found in the whole ischemic territory, whereas a CIS of 3 signifies that the whole ischemic area exhibits capillary blush. CIS 2 or 3 (≤1/3 of the ischemic area has no capillary blush) is considered favorable CIS (*f* CIS) and it was found to be a *prerequisite for a GCO* in BMC-AIS registry (61). A CIS of 0 or 1 was considered a poor score CIS (pCIS), and no patients with *p*CIS had a GCO despite good revascularization (61). The merit of the CIS as a method for patient selection was further validated in a recent IMS I and II subgroup analysis (20). Of patients with *f* CIS and good revascularization (mTICI, score 2b or 3), 100% achieved GCO, while patients with *p*CIS invariably did worse than the natural history of the disease estimated at 25% GCO, as shown by the PROACT II study, independent of revascularization status (Table 2) (3). Recently, we applied the CIS to a subgroup of IMS III cohort and found almost identical findings (Al-Ali, Firas et al. Relative Influence of Capillary Index Score, Revascularization and Time on Stroke: Outcomes from the IMS III trial. Submitted to *Stroke* February 2015).

**Table 2:** **CIS vs. Outcome**.

	*f* CIS:% of mRS, 0–2	*p*CIS:% of mRS, 0–2
BMC-AIS (TIMI 0,1)	0	0
BMC-AIS (TIMI 2,3)	60	0
BMC-AIS (TIMI 3)	83	0
IMS I, II (TIMI 0,1)	33	0
IMS I, II (mTICI 2,3)	86	13
IMS I, II (mTICI 2b,3)	100	20

The percentage of *f* CIS, which was a prerequisite for GCO, was found to be 42% in the BMC-AIS registry and 46% in the IMS I, II subgroup analysis (20, 57), all hovering around 50%, which strengthens our belief in “the 50% barrier” hypothesis.

## Ischemic Territory vs. the Site of Vascular Occlusion

**Figure 8:**
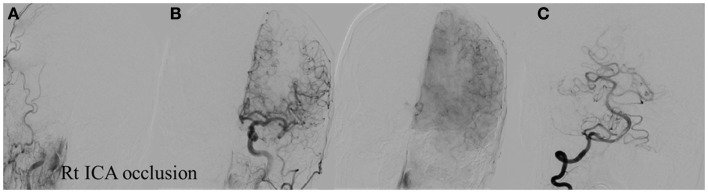
**Vascular occlusion and ischemic territory**. This patient has occlusion of the right ICA and a congenital absence of the anterior and posterior communicating arteries (Acom, Pcom). The resulting ischemic territory is the right MCA and ICA. **(A)** Frontal view of the injection of the right common carotid artery demonstrating no intracranial capillary blush. **(B)** Frontal view of the injection of the left common carotid artery demonstrating no collateral flow to the right hemisphere through the interior cerebral artery. **(C)** Frontal view of the injection of the right vertebral artery demonstrating no collateral flow to the right interior carotid artery territory.

**Figure 9:**
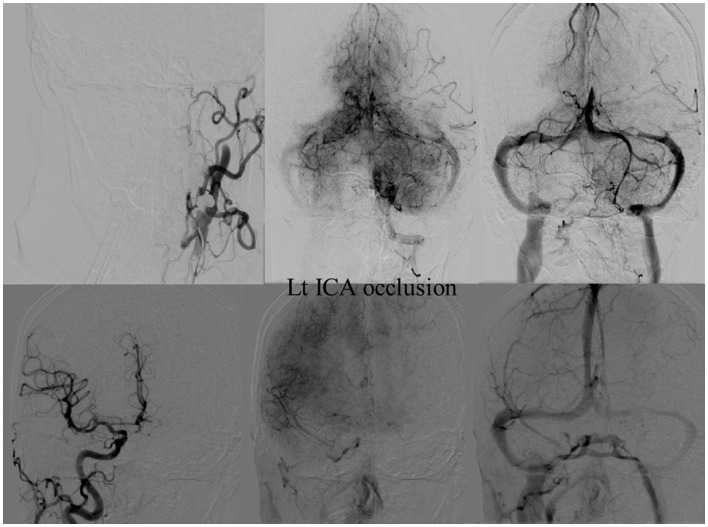
**Vascular occlusion and ischemic territory**. This patient has occlusion of the left internal carotid artery (ICA), but has a well-developed anterior communicating artery (Acom). The resulting ischemic territory is only the left MCA territory.

Central to the concept of CIS is the concept of *ischemic territory*. We define ischemic stroke by the ischemic territory instead of the site of vascular occlusion since we believe that it gives a more accurate estimation of stroke extension. The *ischemic territory* is defined as the *area of the brain that lacks antegrade flow. All or a portion may receive its blood supply in retrograde fashion through pial collaterals*. For example, in a patient with internal carotid artery (ICA) occlusion, a few radically different scenarios are possible. In one scenario, the patient has congenital absence of the Acom and the Pcom arteries (Figure 8). This patient’s ischemic territory will include the entire ipsilateral middle and anterior cerebral artery territories. In a different scenario with the exact same ICA occlusion, but with Acom artery present and well-developed, the anterior cerebral artery territory ipsilateral to the vascular occlusion will receive an antegrade blood supply from the counter lateral ICA, through the patent Acom artery so the ischemic territory will encompass only the MCA territory (Figure 9). Hence, due to multiple possible scenarios when using the site of vascular occlusion to describe the ischemic stroke, we believe that defining the stroke by its territory is a more accurate approach.

## The CIS Limitation

The main limitation of the CIS is the need to perform a full DCA during intervention. However, we believe the significant information obtained through the CIS by examining the DCA, mainly how to guide patient selection combined with the elimination of an arbitrary time window greatly outweighs the minimal risk associated with adding a few injections for a required DCA during intervention.

## CIS vs. Non-Invasive Testing

Interestingly, the *f* CIS and *p*CIS groups had almost identical values concerning time from stroke onset, the ASPECT score, and the National Institutes of Health Stroke Scale (NIHSS) scores in the BMC-AIS registry and the IMS I, II subgroup analysis (20, 61). The relationship between CIS and different perfusion imaging parameters was evaluated in the DEFUSE II trial. Although there was a good general agreement between the CIS score and the time to maximum values >6 and >10 (Tmax > 6 and Tmax > 10), low CIS correlated with high Tmax > 6. There was a significant overlap between the different CIS and the Tmax values, which makes it impossible to differentiate between the *f* CIS and *p*CIS based solely on the MRI perfusion parameter. (Oral presentation at the International Stroke Conference, San Diego, CA, USA, February 2014). These findings imply that the CIS provides different information than currently available from non-invasive tests, which cannot be extrapolated with confidence. Furthermore, none of these non-invasive tests has a similar threshold to the CIS (*f* vs. *p*CIS) that can be used confidently in patient selection. As shown, even with the most useful non-invasive test today, MRI diffusion/perfusion imaging, only 50% of patients who achieve good revascularization have a GCO (49).

## The Relative Importance of CIS, Revascularization, and Time

Several important observations were made after applying the CIS retrospectively on different registries and trials [(20, 61), Al-Ali, Firas et al. Relative Influence of Capillary Index Score, Revascularization and Time on Stroke: Outcomes from the IMS III trial. Submitted to *Stroke* February 2015]. First, *f* CIS was almost a pre-requisite for GCO following revascularization. In other words, when good revascularization (TIMI 2, 3) was achieved on patients with *p*CIS, it was futile (no clinical improvement). Revascularization mattered *only* when patients had excellent collaterals, as indicated by *f* CIS. Next, and despite the fact that *f* CIS was almost a prerequisite for GCO, its presence alone was not sufficient to guarantee GCO. In the IMS I and II, patients with *f* CIS had 100 vs. 38% GCO with or without good recanalization, respectively (20). These observations demonstrate the concomitant importance of recanalization and the fact that *f* CIS is an indicator of ischemic but contemporaneously viable tissue, but not an indicator of perpetually viable tissue. Recanalization is still required. If these observations are supported in a prospective trial, it may significantly change the AIS treatment algorithm, where IAT could be offered to all patients with *f* CIS but not patients with *p*CIS, regardless of time of ictus. This will constitute a radical shift of the present approach and liberate the decision making from an arbitrary time window, in favor of a more physiological basis.

## Proposed Patient Selection Algorithm

We recognize that the merit of the CIS still needs to be proven in a multicenter prospective study; however, we believe the CIS hypothesis will be proven true due to its ability to explain the results of ischemic stroke trials.

**Figure 10:**
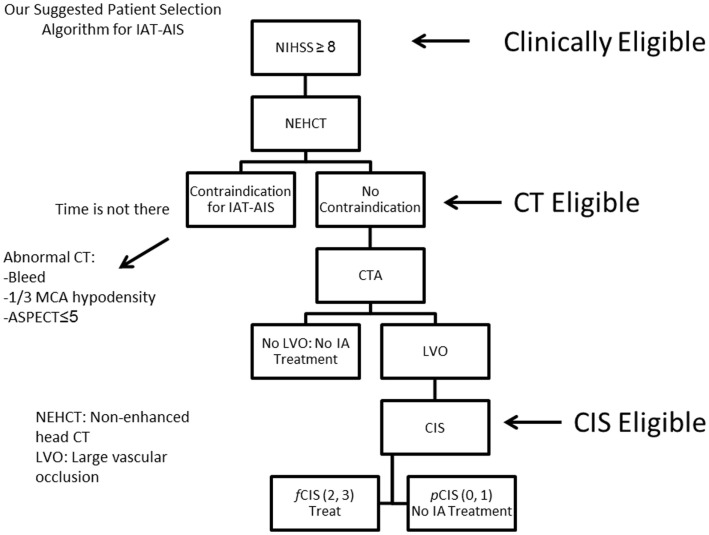
**Proposed patient selection algorithm for AIS**.

Since most patients will improve to a variable degree with time and physical therapy, we believe that IAT should be offered to patients suffering from a large stroke (NIHSS < 8), with the only exception being aphasia (Figure 10). Of those patients who are *Clinically Eligible*, a non-enhanced head CT is obtained followed by CTA. These non-invasive tests can first rule out stroke mimics and identify patients with already visible signs of structural changes due to large irreversible ischemia (i.e., hypodensity in >1/3 MCA territory on head CT). If no such findings are identified, CTA will help confirm the vascular occlusion and its location. Patients with no counter-indication to treatment and proven large vessel occlusion (*CT Eligible)* are offered IAT. A full DCA is performed on these patients to obtain the CIS. Only patients who demonstrate *f* CIS (*CIS Eligible*) should be offered IAT since revascularization on patients with *p*CIS will be futile and possibly harmful (Figure 10). If these steps are taken, we predict a significant increase in the ratio of GCOs between treated and untreated patients on the order of 5–6, instead of the current 1.6–1.7 ratio that exists currently (3, 10–13), by the virtue of significantly decreasing the percentage of futile and harmful revascularization.

## Conclusion

The current approach for treating AIS is based on arbitrary time windows and revascularization, but we believe collaterals also need to be taken into account. We argue that only approximately 50% of all patients with AIS have robust enough collaterals to permit GCOs following treatment, a concept we call *the 50% barrier*. Previous and ongoing genetic work should shed light on this interesting possibility in the near future. The CIS can identify patients with viable tissue, who are therefore candidates for treatment, and dispose of the arbitrary time window.

## Conflict of Interest Statement

The authors declare that the research was conducted in the absence of any commercial or financial relationships that could be construed as a potential conflict of interest.
